# Gene expression profile of cervical and skin tissues from human papillomavirus type 16 E6 transgenic mice

**DOI:** 10.1186/1471-2407-8-347

**Published:** 2008-11-26

**Authors:** D Mendoza-Villanueva, J Diaz-Chavez, L Uribe-Figueroa, C Rangel-Escareão, A Hidalgo-Miranda, S March-Mifsut, G Jimenez-Sanchez, PF Lambert, P Gariglio

**Affiliations:** 1 Departamento de Genética y Biología Molecular, Centro de Investigación y de Estudios Avanzados, México DF 07000, México; 2 Instituto Nacional de Medicina Genómica, DF, México; 3 McArdle Laboratory for Cancer Research, University of Wisconsin School of Medicine and Public Health, Madison, Wisconsin 53706, USA

## Abstract

**Background:**

Although K14E6 transgenic mice develop spontaneous tumors of the skin epithelium, no spontaneous reproductive tract malignancies arise, unless the transgenic mice were treated chronically with 17β-estradiol. These findings suggest that E6 performs critical functions in normal adult cervix and skin, highlighting the need to define E6-controlled transcriptional programs in these tissues.

**Methods:**

We evaluated the expression profile of 14,000 genes in skin or cervix from young K14E6 transgenic mice compared with nontransgenic. To identify differentially expressed genes a linear model was implemented using R and the LIMMA package. Two criteria were used to select the set of relevant genes. First a set of genes with a Log-odds ≥ 3 were selected. Then, a hierarchical search of genes was based on Log Fold Changes.

**Results:**

Microarray analysis identified a total of 676 and 1154 genes that were significantly up and down-regulated, respectively, in skin from K14E6 transgenic mice. On the other hand, in the cervix from K14E6 transgenic mice we found that only 97 and 252 genes were significantly up and down-regulated, respectively. One of the most affected processes in the skin from K14E6 transgenic mice was the cell cycle. We also found that skin from transgenic mice showed down-regulation of pro-apoptotic genes and genes related to the immune response. In the cervix of K14E6 transgenic mice, we could not find affected any gene related to the cell cycle and apoptosis pathways but did observe alterations in the expression of immune response genes. Pathways such as angiogenesis, cell junction and epidermis development, also were altered in their gene expression profiles in both tissues.

**Conclusion:**

Expression of the HPV16 E6 oncoprotein in our model alters expression of genes that fell into several functional groups providing insights into pathways by which E6 deregulate cell cycle progression, apoptosis, the host resistance to infection and immune function, providing new opportunities for early diagnostic markers and therapeutic drug targets.

## Background

Cancer development usually takes several decades to arise, and follows a progressive histopathological pattern that involves acquisition of multiple genetic changes to the cancer cell. Human papillomaviruses (HPVs) are small DNA tumor viruses that cause benign tumors in human skin. A subset of anogenital HPVs, the high-risk HPVs (HR-HPVs), is associated with human malignant tumors, including the majority of cervical cancers [1]. HPV-associated cervical carcinogenesis is a multistep process, in which infected cells develop into cervical intraepithelial neoplasia (CIN) and then into malignant cancer [2]. Two genes of HR-HPVs, E6 and E7, are expressed in the cells derived from HPV-associated cancers [3,4]. Viral oncoproteins are multifunctional proteins that cooperate with each other [5,6] and with other oncogenes [7-10] in the immortalization or transformation of cells. The transforming activities of E6 and E7 correlate, at least in part, with the inactivation of two cellular tumor suppressor gene products, p53 and pRb, which regulate the processes of cell division, differentiation, and/or death [11-14].

The *in vivo *properties of HR-HPV E6 and E7 oncoproteins have been evaluated through the generation and characterization of HPV transgenic mouse strains [15-17]. In the context of the K14E6 and K14E7 transgenic mice, expression of the E6 and E7 genes of the HR-HPV type 16 (HPV16), respectively, has been directed to the basal layer of the stratified epithelium, including the cervical epithelium [15,16]. Although K14E6 and K14E7 mice develop spontaneous tumors of the skin epithelium, no spontaneous reproductive tract malignancies arise [15,16], unless the transgenic mice were treated chronically with 17β-estradiol [18-21]. Interestingly, skin tumors derived from E6 were mostly malignant, as opposed to the tumors from E7 mice which were usually benign [15], suggesting that E6 contributes differently than E7 to HPV-associated carcinogenesis. When treated with exogenous estrogen for 6 months, 100% of E7 transgenic mice developed cancer throughout the reproductive tract, but E6 transgenic mice did not. E6 oncogene synergizes with estrogen to induce cervical cancer in only 41% of K14 E6 mice after 9 months, indicating that E6 has a weaker but detectable oncogenic potential in the cervix compared with the E7 oncogene [22]. It is known that E6's inactivation of p53 contributes to mouse cervix transformation [22,23], but the ability of E6 to promote cell proliferation in K14E6 mice seemed to be p53 independent, as the epidermis from p53-knockout mice did not display an increase in the BrdU labeling index in skin [15], as compared with that of nontransgenic mice. In contrast, the epidermis from K14E6/p53-null mice display epithelial hyperplasia, suprabasal DNA synthesis or cell differentiation inhibition [15]. These indicate that in K14E6 mice, E6 activities other than its inactivation of p53 contribute to its induction of epithelial hyperplasia. Additionally, some mutant E6 proteins that are unable to inactivate p53 retain the ability to transform cells [24-26] or induce several phenotypes in vivo [27]. Conversely, other mutant E6 proteins that retain the ability to target p53 for degradation are unable to induce transformation [25-28]. These observations suggest that other E6-interacting proteins might contribute to important phenotypes induced by E6. A particularly intriguing group of proteins is the PDZ domain proteins. High-risk but not low-risk HPV E6 proteins associate and destabilize PDZ domain proteins [29]. In the context of mice, the ability of E6 to bind PDZ proteins correlates with its ability to induce cancers both in the context of the skin [30] and the cervix [22]. Taken together, these findings suggest that E6 performs critical functions in normal cervix or skin and highlight the need to define E6-controlled transcriptional programs in these tissues.

In the present study, we evaluated the expression profile of 14,000 genes in K14E6 skin or cervix compared with corresponding tissues from nontransgenic mice (FVB mice). Overall, 676 genes were up-regulated and 1154 genes were down-regulated in the skin, while 97 genes were up-regulated and 252 genes were down-regulated in the cervix. Differences in gene expression patterns between FVB and K14E6 transgenic mice indicated a role for E6 in keratinocyte proliferation, cell adhesion, motility, apoptosis and differentiation, which might represent early steps in E6 induced carcinogenesis. This investigation is the first to attempt a comprehensive description of pathways altered by HPV16 E6 alone in transgenic mice.

## Methods

### Mice and RNA isolation

K14E6 transgenic mice have been described previously [15]. For each microarray analysis three FVB and three K14E6 transgenic 6-week-old virgin female mice were employed. All mice were sacrificed and shaved with razor. Mice were housed and treated according to the American Association of Laboratory Animal Care (AALAC) regulations. All mouse procedures were performed according to a protocol approved by the Research Unit for Laboratory Animal Care Committee (UPEAL-CINVESTAV-IPN, Mexico; NOM-062-ZOO-1999). After sacrifice dorsal skin (approx. 1 cm^2^) and lower reproductive tract (cervix and vagina) were removed and immediately frozen in liquid nitrogen for later RNA isolation. Collected tissues were homogenized by mortar in liquid nitrogen, total RNA was extracted using TRIZOL reagent (InVitrogen) and purified using RNeasy Mini Kit (Qiagen) according to the manufacturer's instructions.

### Complementary RNA (cRNA) labeling and hybridization for microarray

The quality and size distribution of the RNA were assessed with the RNA Nano Lab on a Chip kit (Agilent Technologies), which yielded RNA integrity numbers (RIN) from 6.5 to 9.2 with a median of 7.9. Total RNA collected from skin or cervical tissue from three female mice of each condition were pooled. Briefly, 3 μg of the total pooled RNA was converted to first-strand cDNA using Superscript II reverse transcriptase primed by a poly(T) oligomer. Second strand cDNA synthesis was followed by an in vitro transcription reaction in which biotinylated CTP and UTP were incorporated to the generated transcripts. The cRNA products were fragmented to 200 nucleotides or less, then, 15 μg of the fragmentation product were used to prepare 300 μl hybridization cocktail (100 mM MES, 1 M NaCl, 20 mM EDTA, 0.01% Tween-20, 0.1 mg ml-1 of HS DNA, and 0.5 mg ml-1 acetylated bovine serum albumine). The cocktails were heated to 95°C and hybridized in the Mouse Genome 430A 2.0 Array (Affymetrix Inc.) for 16 hours at 45°C. After hybridization, arrays were washed at low (6 × SSPE) and high (100 mM MES, 0.1 M NaCl) stringency and stained with streptavidin-phycoerythrin. Fluorescence was amplified by adding biotinylated anti-streptavidin and an additional aliquot of streptavidin-phycoerythrin stain. The GeneChip Scanner 3000 7G (Affymetrix, Santa Clara CA) was used to collect fluorescence signal of 11 um feature size resolution after excitation at 570 nm. GCOS software (Affymetrix, Santa Clara CA) was used to obtain intensity signal and quality data of the scanned arrays.

### Statistical analysis

Each microarray experiment was repeated as technical replicates for statistical robustness. Data preprocessing included two normalization processes: quantile normalization [31] was applied to technical replicates and then Loess normalization applied to all microarrays to standardize the dynamic range of expression levels. To identify differentially expressed genes a linear model was implemented using R and the LIMMA package [32]. The microarray data were deposited MIAME compliant to NCBI GEO database [GEO: GSE10702]. Two criteria were used to select the set of relevant genes. First a set of genes with a Log-odds ≥ 3 were selected. Then, a hierarchical search of genes was based on Log Fold Changes.

### Analysis of array data

To identify those biological processes that show differentially expressed genes, we used the MAPPFinder analysis [33] on this dataset, using a cutoff ≥ 1.7 in gene expression for both tested tissues (see Additional file 1). For further biological meaning of changes in gene expression, genes examined were submitted to the visualization tool GeneMAPP (see Additional file 2). This bioinformatic tool is employed for visualizing expression data in the context of KEGG biological pathways.

### Real-time RT-PCR

Isolated RNA was controlled for quality by 2% agarose gel separation and ethidium bromide staining. RNA was quantified by spectrophotometry. Complementary DNA (cDNA) was synthesized using 2 μg of total RNA. The 20 μl reverse transcription reaction consisted of 2 μl 10× RT buffer, 0.5 mM each dNTP, 1 μM Oligo-dT primers, and 4 U Omniscript reverse transcriptase (QIAGEN, USA). The reverse transcription reaction was incubated for 1 h at 37°C and then at 93°C for 15 min. A no-template control was performed for each experiment, establishing the absence of genomic contamination in the samples. For the quantitative SYBR Green real-time PCR, 1 μl of each RT product was used per reaction and SYBR Green reaction was conducted using a QuantiTect™ SYBR Green PCR Reagents kit (QIAGEN, USA) and the protocol provided by the manufacturer. Optimization was performed for each gene-specific pair of primers prior to the experiment to confirm that 50 nM primer concentrations did not produce nonspecific primer-dimmer amplification signal in no-template control tube. Changes in fluorescence were recorded as the temperature was increased from 65°C to 95°C at a rate of 0.2°C/s to obtain a DNA melting curve. The characteristic peak at the melting temperature of the target product distinguishes it from amplification artefacts that melt at lower temperatures in broader peaks. The primer sequences, that were designed using Primer Express Software, confirmed specificity of the PCR (Table 1). Each sample was tested in triplicate with quantitative PCR and, for standardization of gene expression levels, mRNA ratios relative to the house-keeping gene GAPDH were calculated. We evaluated mRNA expression in a total of 3 mice from each group.

**Table 1:** Primer sequences for quantitative RT-PCR

**Gene Title**	**Forward primer 5'-3'**	**Reverse primer 5'-3**
TNF receptor superfamily member (Fas)	TTGGAAAATCAACCCCAGACA	TGGCAGGCTCTCTCCTCTCTT
Baculoviral IAP repeat-containing 5 (Birc5)	TCCACTGCCCTACCGAGAAC	TGCTCCTCTATCGGGTTGTCA
Caspase 8 (Casp8)	GGCAGGCTTCGAGCAACA	CGTAGCCATTCCCAGCAGAA
Cyclin E2 (Ccne2)	GCTGCCGCCTTATGTCATTT	AAGGCACCATCCAGTCTACACA
Cyclin-dependent kinase 4 (Cdk4)	TTTCTAAGCGGCCTGGATTTT	CCAGCTTGACGGTCCCATTA
Claudin 4 (Cldn4)	TCATCGGCAGCAACATCGT	TCGTACATCTTGCACTGCATCTG
Gap junction membrane channel protein beta 6 (Gjb6)	GCTTCATTTCGAGGCCAACT	AGGTAACACAACTCGGCCACAT
Matrix metallopeptidase 11 (Mmp11)	TGGAACTCAGGCCAAAAGGT	GGGCAAGGCTGTGAGGTATG
Tight junction protein 2 (Tjp2)	ATTCTCAAGATCAACGGCACTGT	TCAACACCACAAGCTGCAGTT

### Data analysis using 2^-ΔΔCT ^method

Real-time PCR was performed on the corresponding cDNA synthesized from each sample. The data were analyzed using the equation described by Livak ()[34] as follows: Amount of target = 2^-ΔΔCT^. We used the average Δ T from Cervix FVB as calibrator for each gene tested. Validation of the method was performed as previously reported [35]. Data are presented as mean ± standard deviation (S.D.). Statistical evaluation of significant differences was performed using the Student's t-test. Differences of P < 0.05 were considered statistically significant.

### Determination of E6 isoforms

E6 RNA structures were determined by primer extension of the RNAs followed by amplification of the cDNA products by PCR. The forward primer for full-length E6 was 5'-ATG TTT CAG GAC CCA CAG GA-3', for E6* was 5'-TAC TGC GAC GTG GAC GTG AGG TGT ATT AAC-3', and for E6** was 5'-TAC TGC GAC GTG AGA TCA TCA-3'. All of them used the same reverse primer 5'-CAG TTG TCT CTG GTT GCA AAT C-3' and the annealing was at 59°C.

## Results

**Figure 1:**
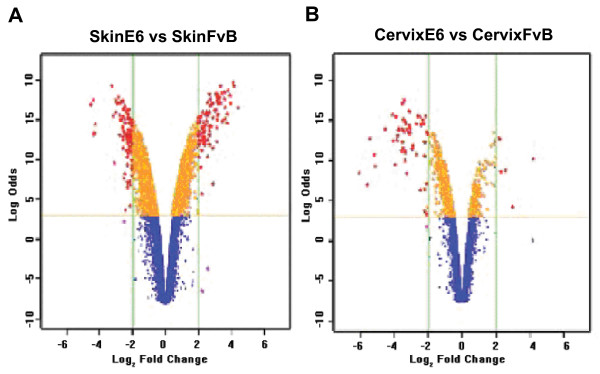
**Volcano Plot for skin and cervix data set**. Volcano plot for the 14000 genes from the GeneChip Mouse Genome data. The x-axis is the Log_2 _Fold-Change value and the y-axis is Log Odds value. In each graph, every point represents an individual transcript. A) Volcano Plot for skin data set. The vertical lines represent 2 fold changes, both up-regulated (right side) and down-regulated (left side) and the horizontal lines represent a Log Odds ≥ 3. B) Volcano Plot for cervix data set. The vertical lines represent 2 fold changes, both up- and down-regulated and the horizontal lines represent a Log Odds ≥ 3 as the threshold cutoff.

**Figure 2:**
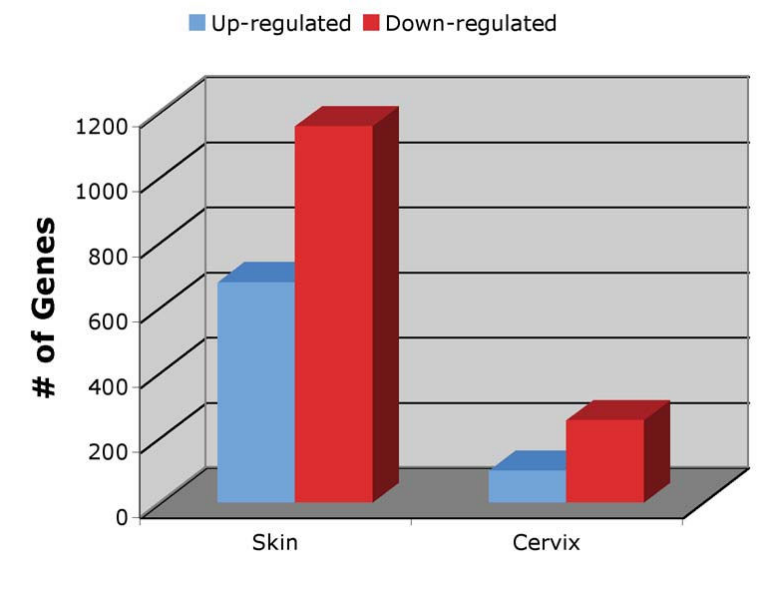
**Gene expression profile in skin and cervix from K14E6 transgenic mice**. Overall, 676 genes were up-regulated, 1154 genes were down-regulated in skin, and 97 genes were up-regulated, 252 genes were down-regulated in cervix; with a Log Odds ≥ 3 and a fold change ≥ 1.7.

In this study our major aim was to identify the gene expression profile in skin or in cervix from K14E6 transgenic compared with corresponding tissues from FVB mice. It has been shown that these tissues have different behavior in presence of the E6 oncoprotein [21]. In order to identify genes involved in the observed differential phenotypes in these tissues, we performed a comprehensive analysis of genome-wide expression with microarrays. We noticed that the number of genes differentially expressed in both tissues was significantly different, as shown in Figure 1. The genes on the upper left or upper right corners of the volcano plot represent large statistically significant changes with large fold changes (Figure 1). To identify genes with statistically significant changes we selected those genes with a Log Odds ≥ 3 and a fold change ≥ 1.7 and ≤ -1.7 as the threshold cutoff. It was found that a total of 1830 genes were differentially expressed in skin from K14E6. Out those, 676 were up-regulated and 1154 down-regulated genes. In contrast, only 349 genes showed differential expression in cervix from K14E6 transgenic mice, 97 up-regulated and 252 down-regulated. Showing a 5-fold difference (Figure 2). One explanation for the different gene expression in skin compared with cervix from K14E6 transgenic mice could be difference in expression of the full-length E6 or E6* and E6** isoforms in those tissues. To determine transgene mRNA expression, we performed semi-quantitative RT-PCR using PCR primers amplifying full-length E6, E6* and E6** isoforms [36]. We did not find any significant change in expression of these isoforms between tissues (Data not shown). A prior study documented that similar levels of full length E6 protein expression are expressed in the lower female reproductive tract and the skin of K14E6 mice as is found in human cervical cancer cell lines [22].

**Figure 3:**
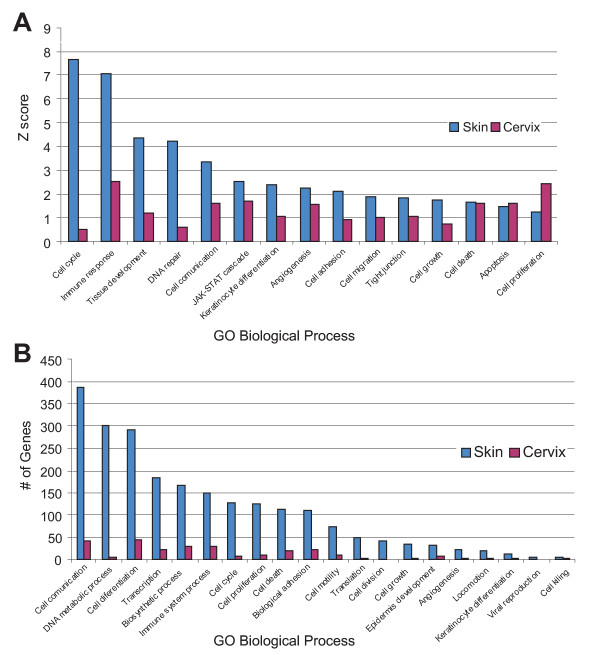
**Gene Ontology-based biological process pathways altered in Skin and Cervix from K14E6 transgenic mice**. To know biological process pathways involved, we imported a list of significant up and down-regulated genes for each tissue to MAPPFinder. This figure is meant to provide an overall view on transcript changes. A) GO biological processes ranked by z score. A z score near zero indicates that the number of genes meeting the criterion approximates the expected number, extreme scores suggest GO terms with the greatest confidence that the correlation between the expression changes of the genes in this grouping are not occurring by chance alone. B) GO biological processes ranked by number of genes affected. Note that because genes may appear multiple times within these hierarchies, the number of genes provided in this figure is relative to a certain biological process, not absolute.

Using significantly up and down-regulated genes, MAPPFinder identified the GO categories altered and we show a representative number of these processes (Figure 3). In addition, MAPPFinder showed that roughly 94% of genes showing altered expression were annotated in GO. Then genes examined were submitted to GeneMAPP in an attempt to identify significantly dysregulated pathways (see Additional file 2). This confirmed that genes involved in several metabolic pathways were altered in both tissues.

**Figure 4:**
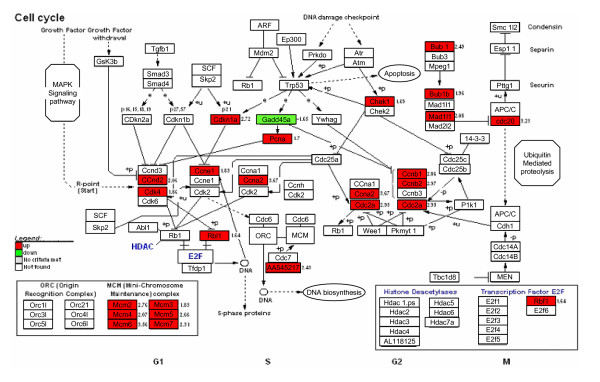
**KEGG-Cellular Pathways integrating our expression data of cell cycle in skin and cervix from K14E6 transgenic mice**. For skin, up-regulated genes are showed in red and down-regulated genes in green. The only gene differentially expressed in cervix from K14E6 mice is shown by a blue arrow, cadherin 1 (Cdh1), which was down-regulated. The numbers represents the fold change of the gene.

**Figure 5:**
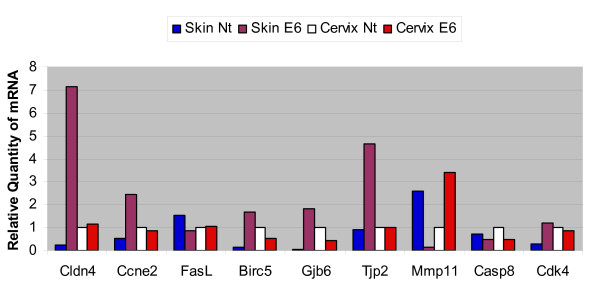
**Real-time RT-PCR**. Validation of selected genes differentially expressed from Skin and Cervix of K14E6 transgenic mice (purple and red bars respectively) and nontransgenic mice (blue and white bars respectively). The results of one experiment are shown and are representative of two separate studies with similar results.

One of the most affected processes in skin from K14E6 transgenic mice was the cell cycle, which shows overexpression of genes that induced cellular proliferation like cyclins A2, E, D2, B1, B2 and Cdk4 (Figure 4). The minichromosome maintenance family (MCM) related to initiation and elongation of replication forks were also up-regulated (Figure 4). On the other hand, cervix from transgenic mice did not show a significant change in the expression of these genes (Figure 4). Expression of Ccne2 (cyclin E) and Cdk4 genes was validated by real time RT-PCR and the results agree with those of microarrays (Figure 5).

**Figure 6:**
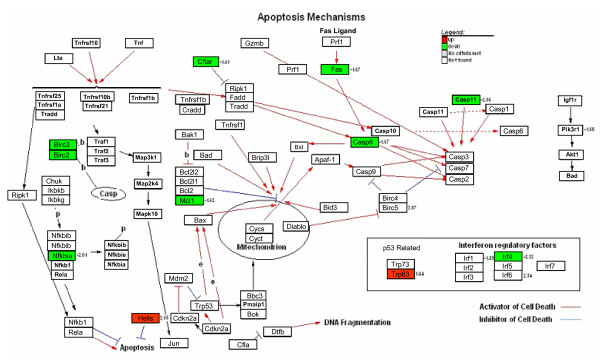
**KEGG-Cellular Pathways integrating our expression data of apoptosis in skin and cervix from K14E6 transgenic mice**. For skin, up-regulated genes are showed in red and down-regulated genes in green. In cervix from K14E6 transgenic mice no effect was observed in this pathway. The numbers represents the fold change of the gene.

It is well known that high risk HPV16 E6 oncoprotein inhibits apoptosis [37], thus we checked if there were alterations in the apoptosis pathway in the tissues from K14E6 mice. We found that skin from K14E6 mice showed down-regulation in the expression of pro-apoptotic genes, particularly in those related to the extrinsic apoptotic pathway like Caspase 8 and Fas (Figure 6). We also observed the increased expression of anti-apoptotic genes like Birc5 (Survivin) and Hells (Figure 6). We validated that the expression of Caspase 8, Fas and Birc5 genes was altered by real time RT-PCR (Figure 5). In cervix from transgenic mice, we could not find any gene related to the apoptosis pathway altered in its expression.

**Figure 7:**
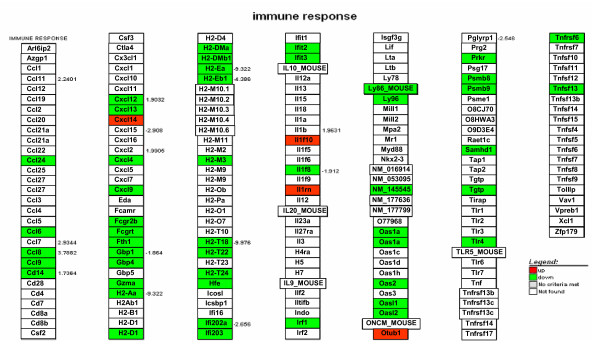
**KEGG-Cellular Diagrams integrating our expression data of immune response in skin from K14E6 transgenic mice**. Up-regulated genes are showed in red and down-regulated genes in green. The numbers represents the fold change of the gene.

**Figure 8:**
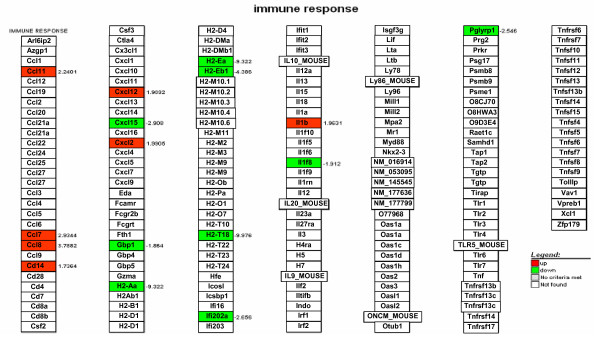
**KEGG-Cellular Diagrams integrating our expression data of immune response in cervix from K14E6 transgenic mice**. Up-regulated genes are showed in red and down-regulated genes in green. The numbers represents the fold change of the gene.

Another pathway that was severely affected in skin of K14E6 transgenic mice was the immune response, showing down-regulation of interferon related genes like Ifi203, Ifi202a, Irf1, and Ifit2 (Figure 7). Similar effects were observed with the 2'-5' oligoA synthetase family (Oas), which mediates RNA decay as part of the innate antiviral immunity pathway, and the major histocompatibility 2 complex (Figure 7). Interestingly, we also observed alterations in immune response genes expression in cervix from K14E6 transgenic mice (Figure 8).

Additional pathways were analyzed like angiogenesis, cell junction, cytoskeleton, keratinocyte differentiation and epidermis development, all of which showed differential gene expression in skin and to a lesser degree the cervix of K14E6 transgenic mice compared to that of nontransgenic mice (see Additional file 2).

## Discussion

It was previously found that expression of HPV16 E6 increased cell proliferation and induced epidermal hyperplasia in K14E6 transgenic mice. Spontaneous skin tumors developed in adult K14E6 mice with an incidence of about 7% at 1 year of age [15]. These tumors were mostly malignant, indicating that E6 alone not only is sufficient to induce benign tumors but may contribute to the development of malignancy in animals [15]. However, expression of E6 oncoprotein induced only low-grade cervical dysplasia without additional neoplastic progression after 6-months of treatment with 17β-estradiol, in contrast to E7, which induced cervical cancer [21]. Only upon extended treatment with estrogen for nine months did K14E6 mice develop cervical cancers [22]. Due to the different properties of the K14E6 transgenic mice in different tissues, we evaluated the expression profile in both the skin and cervix of these mice compared to that of the corresponding tissues from FVB mice.

It is important to notice that we used young mice without apparent lesions, so our results may reflect only the early or direct effects of E6 on gene expression profiles in skin or cervix. We believe that initial steps in carcinogenesis might be crucial in cancer development. Due to the high number of pathways affected by E6 expression, particularly in skin (see Additional file 1), it is difficult to discuss each in detail, but it is worthwhile to point out several interesting observations that our results have provided.

Cyclins are a family of proteins that control the cell cycle by associating with cyclin-dependent kinases (cdks). Cyclin B1 appears at S phase, peaks in expression at G2/M, and it is rapidly degraded at the end of mitosis by ubiquitination and targeting to the proteasome [38]. Studies in different human tumors and cell lines including breast, lung, colorectal, lymphoma, leukemia, and melanoma have detected increased levels of cyclin B1 at both protein and mRNA [39]. In agreement with these observations, a high differential expression of cyclin B1 was consistently found in skin from K14E6 as compared to nontrangenic mice, but we did not see significant modulation of cyclin B1 or other components of the cell cycle control in cervix from K14E6 mice. The abnormally increased cyclin B1 level previously found in human tumors has been shown to correlate with either mutation or deletion of p53 function [40]. Thus, it is likely that the inactivation of p53 function by HPV16 E6 oncoprotein might be responsible for cyclin B1 overexpression in skin. We also observed up-regulation in E2F-responsive genes belonging to the MCM family, involved in DNA replication [41], and in cyclin E, involved in the G1-S transition [42]. Both of these genes were found to be induced by E6 at the protein level by immunohistochemical staining in the same mouse model [22]. In that study MCM7 and cyclin E proteins were found to be elevated in the epithelium lining the K14E6 mouse cervix and vagina. Forkhead box (FOX) proteins constitute an extensive family of transcription factors, which share homology in the winged helix DNA binding domain [43]. Elevated FOXM1 levels have been found in numerous human tumors [44-46], suggesting that FOXM1 is required for cellular proliferation in human cancer cells. In our analysis, the FOXM1 gene was found up-regulated in skin from K14E6 as compared to nontransgenic mice, but we did not find significant modulation of Forkhead box family in cervix from K14E6 mice. Because FOXM1 overexpression in human keratinocytes has been suggested to contribute to cell transformation leading to the development of basal cell cancer [47], it seems likely that FOXM1 overexpression may contribute to HPV-induced keratinocyte transformation and the development of skin cancer.

Topoisomerase II (TOP2A) is a nuclear enzyme that modulates DNA topology during several metabolic processes and is required for the segregation of daughter chromosomes at the end of replication [48]. In our study, TOP2A gene was found differentially expressed in skin from K14E6 as compared to nontrangenic mice. We did not observe significant modulation of TOP2A gene expression in cervix from K14E6 mice. In a previous work it has been shown by microarray analysis in primary HPV16 and HPV18-infected cervical cancers and normal cervical epithelium similar results for genes related to cell cycle control [49]. Interestingly, in another study even the authors found that HPV-positive head and neck cancers (HNCs) and cervical cancers differed in their patterns of gene expression, these tumors shared many changes compared with HPV-negative HNCs, particularly in cell cycle-related genes. For example, HPV-positive cancers, compared to HPV-negative, upregulated a much larger set of cell cycle-specific genes such cyclin E2 (G1 associated), cyclin B1 (G2 associated) and multiple MCMs [50]. An important finding of our study is that the same group of cell cycle-related genes are upregulated by E6 from HPV16 which indicate that our results are consistent with data obtained from human tissues.

Attenuated or diminished apoptosis due to inhibition of Caspases has been implicated as an important mechanism for the onset of tumorigenesis in several cancer types [51,52]. Our results showed that expression of Caspase 8 is lower in skin from K14E6 as compared to FVB mice. We also found down-regulation of Fas, suggesting an alteration in the extrinsic apoptotic pathway. These results are congruent with the differential apoptosis previously observed by other researchers in cervical tumors [53] and cell lines [54,55]. Therefore, alteration or defects in the expression of Caspases and Fas might be a hallmark of skin carcinoma, as it has been observed in several tumor types [51].

In addition, this analysis identified genes that have been associated with the regulation of the immune system. For example, the IFN receptor subunit 2 (IFNAR2), that stimulates transducers and activators of transcription (JAK/Stat) signaling [56], and Stat-1, a primary regulator of the interferon-responsive pathway, were found down-regulated in skin from K14E6 transgenic mice. The IFN-α-JAK/Stat signaling pathway is critical for host defense against viral infection by stimulating transcription of antiviral, antiproliferative, and antitumor genes, suggesting that down-regulation of IFNAR2 and Stat-1 in skin from K14E6 transgenic mice might be critical for immune evasion and therefore for cancer development. It has been reported that E6 from HPV16 binds to interferon regulatory factor-3 interfering with its activity [57], that could explain why we observed down-regulation of many Interferon-inducible genes such as myxovirus (influenza virus) resistance 1 (Mx1), Oas2 and Stat-1. These results agree with another study which used microarray analysis to examine the effect of HPV31 genome on the transcription of cellular genes. They found that an important set of interferon-responsive genes were downregulated by HPV31 [58], suggesting that different HR-HPV types share the mechanism to evade the immune response. Additionally, in cell lines evaluated by microarray analysis it was shown that E6 or E7 from HPV16 can downregulate genes involved in the immune response [59].

On the other hand, it has been demonstrated by others that HPV16 E6 inhibits serum and calcium-induced differentiation of human keratinocytes [60]. Our results identify several of the genes that may be involved in this process (see Additional file 2). A previous study using microarrays showed evidence that E6 and E7 may affect differentiation through downmodulating the transforming growth factor-β (TGF-β) pathway [61]. Interestingly, we also observed downregulation of TGF-β 2 and TGF-β RII mRNA expression in K14E6 transgenic mice. Another group has also shown by microarray analysis that genes involved in keratinocyte differentiation like Small proline-rich proteins family and cytokeratins can be downregulated by E6 oncoprotein [62]. We observed that some of these proteins were downregulated in cervix and for unknown reasons upregulated in skin from K14E6 mice, perhaps related to tissue microenvironment. Nevertheless, both studies indicate that HPV16 modulates expression of differentiation-associated genes. In addition, it has been observed in HPV16 E6 transfected human colon adenocarcinoma cells and human lung adenocarcinoma cells different genomic and proteomic expression patterns [63], in agreement with our results in cervix versus skin from K14E6 mice.

In summary, we identified important genes that are altered at the transcriptional level by E6 in our model, in particular the uncoupling of cell cycle regulation (cdc2, cyclin B, cdk2 and cyclin E), down-regulation of pro-apoptotic genes (Fas) and up-regulation of anti-apoptotic genes (Birc5). In addition, we also observed down-regulation of genes involved in the innate antiviral response such as IFNAR2, Stat-1 and numerous interferon-stimulated genes (Ifi203, Ifi202a, Irf1, Ifit2, Mx1 and OAS). All these changes indicate that E6 could contribute to initiation of carcinogenesis through over-expression of cell proliferation genes and down-regulation of immune response genes, in agreement with the results obtained in a microarray analysis of cervical low grade lesions which are characterized by a pro-proliferative/immunosuppressive gene expression signature [64]. However, E6 is not enough for cancer development and additional factors are necessary in tumor progression such as estrogens, diet and carcinogens [17,21,65,66].

We also observed differentially expressed genes from other pathways like angiogenesis, cell junction, cytoskeleton, keratinocyte differentiation and epidermis development, when skin or cervix from K14E6 transgenic mice were compared with nontransgenic mice; however, we did not discuss these genes in detail in this study. Further investigation may be necessary in skin and cervix from transgenic mice to understand the mechanisms by which E6 affects these pathways.

The results reported here represent the first comprehensive microarray analysis to assess the consequences of HPV16 E6 oncoprotein alone in the normal tissue environment from K14E6 transgenic mice.

## Conclusion

Expression of the HPV16 E6 oncoprotein in our model alters expression of genes that fell into several functional groups providing insights into pathways by which E6 deregulate cell cycle progression, apoptosis, the host resistance to infection and immune function, providing new opportunities for early diagnostic markers and therapeutic drug targets.

## Abbreviations

HPVs: Human papillomaviruses; HR-HPVs: High-Risk HPVs; CIN: Cervical Intraepithelial Neoplasia; FVB: Friend virus B-type susceptibility; HS-DNA: Herring Sperm DNA; MES: 2-(N morpholino)ethanesulfonic acid; MIAME: Minimum Information About a Microarray Experiment; GeneMAPP: Gene MicroArray Pathway Profiler; GO: Gene Ontology; KEGG: Kyoto Encyclopedia of Genes and Genomes.

## Competing interests

The authors declare that they have no competing interests.

## Authors' contributions

DMV and JDC organized sample collection, processing and bioinformatics. LUF performed the microarray experiments. CRE performed statistical analysis. AHM, SMM and GJS participated in aspects of the study design and contributed to the discussion of the results. PFL helped drafting the manuscript. PG is the principal investigator and was involved in the conceptualization, discussion and writing of the manuscript. All authors read and approved the final manuscript.

## Pre-publication history

The pre-publication history for this paper can be accessed here:



## Supplementary Material

Additional file 1 **Table S1**. List of genes with a significant altered gene expression. Click here for file

Additional file 2 **Table S2-S9**. List of genes containing KEGG pathways. Click here for file
